# Associations Between an Individualized Heart Rate-Based Training Index and Mood States in Elite Female Kendo Athletes: An Exploratory Field Study

**DOI:** 10.3390/sports14100451

**Published:** 2026-10-12

**Authors:** Akari Kitahara, Shinsuke Tamai, Shoko Nomura, Rina Kagawa, Hideo Suzuki, Hideo Tsurushima, Koichi Watanabe

**Affiliations:** 1 Doctoral Program in Sports Medicine, Degree Programs in Comprehensive Human Sciences, Graduate School of Comprehensive Human Sciences, University of Tsukuba, Tsukuba 305-8577, Ibaraki, Japan; akarikit111@gmail.com; 2 Institute of Human Sciences, University of Tsukuba, Tsukuba 305-8577, Ibaraki, Japan; tamai.shinsuke.kb@u.tsukuba.ac.jp; 3 Acupuncture and Physical Therapy Teacher Training School, University of Tsukuba, Bunkyo-ku, Tokyo 112-0012, Japan; 4 Department of Sports Sciences, Japan Institute of Sports Sciences, Kita-ku, Tokyo 115-0056, Japan; 5 Faculty of Rehabilitation, R Professional University of Rehabilitation, Tsuchiura 300-0032, Ibaraki, Japan; nomura@u.a-ru.ac.jp; 6 Artificial Intelligence Research Center, National Institute of Advanced Industrial Science and Technology, Tsukuba 305-8560, Ibaraki, Japan; kagawa-r@aist.go.jp; 7 Tsukuba Endoscopy Clinic, Tsukuba, Ibaraki 305-0821, Japan; hideoszk@tsukuba-naishikyo.com; 8 Institute of Medicine, University of Tsukuba, Tsukuba 305-8574, Ibaraki, Japan; hideo-tsurushima@gwe.md.tsukuba.ac.jp; 9 Department of Neurosurgery, Hitachinaka General Hospital, Hitachinaka 312-0057, Ibaraki, Japan; 10 Institute of Health and Sport Sciences, University of Tsukuba, Tsukuba 305-8574, Ibaraki, Japan

**Keywords:** athlete monitoring, fatigue, wearable devices, rating of perceived exertion, Profile of Mood States, elite athletes, kendo

## Abstract

Athlete monitoring commonly includes subjective questionnaires such as the Profile of Mood States, which may be influenced by individual perceptions and situational factors. This one-week exploratory field study examined associations between Persist, an individualized wearable-derived behavioral–physiological measure, and mood states in seven elite female kendo athletes. Before the monitoring period, the heart rate corresponding to the point at which each athlete perceived exercise intensity as “somewhat hard” was individually determined using the rating of perceived exertion. Heart rate and training duration were recorded during kendo training sessions over one week using a wrist-worn wearable device. Mood states were assessed using the Profile of Mood States 2nd Edition (POMS 2). Persist showed a negative association with Fatigue–Inertia in the unadjusted analysis (*r*_s_ = −0.847, *p* = 0.016), but this association did not remain statistically significant after Holm correction for multiple comparisons (adjusted *p* = 0.162). These preliminary findings indicate a possible association between Persist and fatigue-related mood states; however, larger repeated-measures studies are required to evaluate its validity and potential utility for athlete monitoring.

## 1. Introduction

To improve competitive performance, athletes need to tolerate an appropriate level of training load. In kendo, heart rate (HR) has been reported to frequently reach approximately 90% of maximum HR during practice, demonstrating that kendo imposes substantial physical demands [1]. High-intensity exercise is generally accompanied by a subjective sense of exertional discomfort. Furthermore, increases in blood cortisol levels and pain-related measures have been observed before and after kendo competitions, suggesting that kendo induces considerable psychological and physiological stress [2]. Excessive training aimed at improving athletic performance can increase the risk of overtraining syndrome [3]. Accordingly, the All Japan Kendo Federation has highlighted the importance of addressing this issue from a sports science and medical perspective [4]. Therefore, maintaining an appropriate balance between training and recovery is essential for conditioning management in kendo athletes. Integrating subjective assessments with physiological and behavioral monitoring remains an important challenge in athlete monitoring.

In sports settings, mood states are commonly evaluated using subjective questionnaires, among which the Profile of Mood States (POMS) is widely utilized [5]. The POMS is a self-report instrument designed to assess affective and mood states across six dimensions: Tension–Anxiety, Depression–Dejection, Anger–Hostility, Vigor, Fatigue, and Confusion. The Profile of Mood States 2nd Edition (POMS 2), which includes revised scale names and the additional Friendliness subscale, was used in the present study. In athlete monitoring, well-conditioned athletes typically exhibit an “iceberg profile,” characterized by high Vigor and low scores on the other mood dimensions, whereas athletes experiencing extreme fatigue may exhibit an “inverse iceberg profile,” marked by low Vigor and elevated scores on the other dimensions [6,7]. Accordingly, POMS has been considered useful for assessing exercise-induced fatigue and detecting overtraining syndrome [8]. Fatigue and training responses are multidimensional and can be characterized using subjective, physiological, and performance-related measures [9]. Subjective self-report measures are sensitive and practically valuable for athlete monitoring [8]; however, they assess perceived states and may provide information that differs from physiological or behavioral measures. Combining complementary sources of information may therefore provide a broader characterization of athletes’ training responses.

Recent advances in wearable devices have made it possible to obtain physiological indicators, such as HR during exercise, noninvasively and continuously. To explore whether wearable-derived physiological and behavioral data are associated with fatigue-related mood states, we focused on HR during exercise in combination with RPE, specifically the duration of exercise performed at or above an individualized HR threshold corresponding to RPE 13 (“somewhat hard”). Previous work has suggested that combining HR during exercise with perceived exertion may provide information relevant to athletes’ psychological responses during training [10]. In the present study, we defined the proportion of exercise time performed at or above an individualized HR threshold corresponding to RPE 13 (“somewhat hard”) as “Persist.” Because Persist may also reflect training-intensity distribution, session content, physical fitness, recovery status, motivation, and other factors affecting exercise behavior, the present study examined its association with fatigue-related mood states without assuming that Persist specifically measures mental condition.

Therefore, the purpose of this study was to explore associations between Persist and mood states during a one-week period of field-based kendo training and to obtain preliminary evidence regarding Persist as a wearable-derived monitoring measure.

## 2. Materials and Methods

### 2.1. Participants

The participants were seven adult female elite kendo athletes. Their competitive characteristics included a kendo grade of 5.3 ± 1.3 Dan, 27.7 ± 5.3 years of kendo experience, and 4.0 ± 1.4 training sessions per week. All participants had competed at the national level, and two had won a national championship title. The participants’ physical and exercise-related characteristics are presented in Table 1.

### 2.2. Study Design

This study was designed as a one-week prospective observational field study. Before the start of monitoring, each participant prepared her own training plan and performed kendo training according to this plan during the monitoring period. HR data recorded using a wearable device were used to calculate the duration of exercise performed at an intensity corresponding to “somewhat hard” or higher. The total duration of such exercise over the one-week period was then divided by the total duration of kendo training, and this proportion was defined as Persist. Mood states were assessed using the Profile of Mood States 2nd Edition (POMS 2). After the monitoring period, participants completed a single retrospective POMS 2 assessment referring to the preceding week; no repeated within-athlete mood assessments were obtained during the monitoring period. The relationships between Persist and each questionnaire score were then examined.

### 2.3. Wearable-Derived Measures and Calculation of Persist

Before field monitoring, a preliminary assessment was conducted at the Tochigi Institute of Sports Medicine and Science between 5:00 and 7:00 p.m. in conjunction with routine physiological testing performed for the athletes, which included VO_2_max assessment. For the present study, participants performed an incremental exercise test using a bicycle ergometer (Fujin-Raijin, OCLabo, Tokyo, Japan), with the workload increased by 45 W every 3 min until exhaustion. Before the exercise test, participants were instructed on how to report RPE to ensure familiarity with the reporting procedure. Perceived exertion was assessed using the Borg 6–20 RPE scale, on which RPE 13 corresponds to “somewhat hard.” RPE was not assessed at fixed intervals or at every stage; instead, participants were instructed to indicate when their perceived exertion reached RPE 13 during the exercise test. HR was recorded using a Polar H10 chest-strap monitor (Polar Electro Oy, Kempele, Finland), and the instantaneous HR recorded when the participant reported RPE 13 was defined as the individualized HR threshold used to calculate Persist. VO_2_max assessment was conducted as part of the routine physiological testing but was not used to determine the individualized HR threshold or to calculate Persist. Each participant completed the preliminary exercise test once. Field monitoring began at least one week after the preliminary assessment, although the interval varied among participants. From the day before the preliminary exercise test, participants were instructed to abstain from alcohol and caffeine and to avoid strenuous exercise. In addition, based on the opinions of a psychiatrist and an orthopedic surgeon, participants were confirmed to have no injuries or pain and no psychological conditions requiring clinical diagnosis.

During the monitoring period, HR and training duration were objectively recorded using a wearable device, whereas the individualized HR threshold used to calculate Persist was anchored to the subjectively reported RPE 13 (“somewhat hard”). Persist should therefore be considered an integrated behavioral–physiological measure rather than a purely objective index. In accordance with a previous study [10], HR and training duration were measured using a wrist-worn wearable device (Polar Ignite; Polar Electro Oy, Kempele, Finland).

Persist was calculated as the proportion of total kendo training time spent at or above the individualized HR threshold corresponding to RPE 13. Participants manually started and stopped recording at the beginning and end of each kendo training session. Recording was paused during long breaks in which participants removed their protective equipment, whereas rest periods during which protective equipment remained on were included in the recorded training duration. Non-kendo physical activity was not included in the calculation of Persist. The wearable device was worn on the left wrist to minimize the possibility of direct impact from the shinai during kendo practice. No missing HR epochs were identified in the recorded training data, and all scheduled kendo training sessions during the monitoring period were successfully recorded. No formal artifact-rejection procedure was applied.

### 2.4. Questionnaires

Subjective measures included the POMS 2, Sense of Coherence (SOC), and self-handicapping (SHC). The POMS 2 was used to assess mood states. After the monitoring period, participants were asked to complete the questionnaire based on their mood states during the previous week. Before completing the questionnaire, participants received instructions on how to respond, and the researchers checked the responses to ensure that there were no missing or incorrect entries. Based on the responses, scores were calculated for Anger–Hostility (AH), Confusion–Bewilderment (CB), Depression–Dejection (DD), Fatigue–Inertia (FI), Tension–Anxiety (TA), and Vigor–Activity (VA). Total Mood Disturbance (TMD) was calculated by subtracting the raw VA score from the sum of the raw AH, CB, DD, FI, and TA scores. T-scores for each scale were used in the analysis.

As secondary exploratory psychological measures, SOC and SHC were assessed before monitoring. SOC was included to characterize stress-coping orientation, whereas SHC was included to characterize self-handicapping tendencies related to anticipated failure. These analyses were exploratory and were not intended to establish the construct validity of Persist. SOC is a psychological construct that reflects an individual’s ability to cope with stressors [11,12], and was assessed using the 13-item Sense of Coherence Scale (SOC-13). SHC refers to behaviors or claims made in advance to attribute possible failure to external factors [13,14], and was assessed using the Self-Handicapping Scale [15].

### 2.5. Statistical Analysis

Descriptive data are presented as the mean ± SD. Statistical analyses were performed using SPSS version 26 (IBM Corp., Armonk, NY, USA). Spearman’s rank correlation analysis was used to examine associations between Persist and each questionnaire score. Because ten correlations were examined, the Holm procedure was applied to control for multiple comparisons. For each correlation, *r*_s_, the two-sided *p*-value, a 95% bootstrap confidence interval, and the Holm-adjusted *p*-value were reported. Bootstrap 95% confidence intervals for Spearman’s rank correlation coefficients were calculated using 10,000 participant-level resamples with replacement using the percentile method. Statistical significance was defined as an adjusted *p*-value < 0.05. Given the exploratory nature and small sample size of the study, the correlation analyses were interpreted as exploratory and hypothesis-generating. As a sensitivity analysis for the association between Persist and Fatigue–Inertia, a leave-one-out analysis was performed by sequentially excluding each participant and recalculating Spearman’s rank correlation coefficient.

## 3. Results

### 3.1. Wearable-Derived Measures

During the one-week monitoring period, the total training duration was 16,422.3 ± 3757.0 s (273.7 ± 62.6 min), of which 2337.6 ± 848.2 s (39.0 ± 14.1 min) were spent at or above the individualized RPE 13 HR threshold. The proportion of exercise time spent at this intensity relative to the total training duration, defined as Persist, was 14.7 ± 6.1%.

### 3.2. Questionnaires

The results of the POMS 2, SOC, and SHC are shown in Table 2. In the POMS 2, the mean T-scores for all subscales, including Total Mood Disturbance (TMD), were within the range of 40–59 points.

### 3.3. Correlation Between Persist and Questionnaire Scores

**Figure 1:**
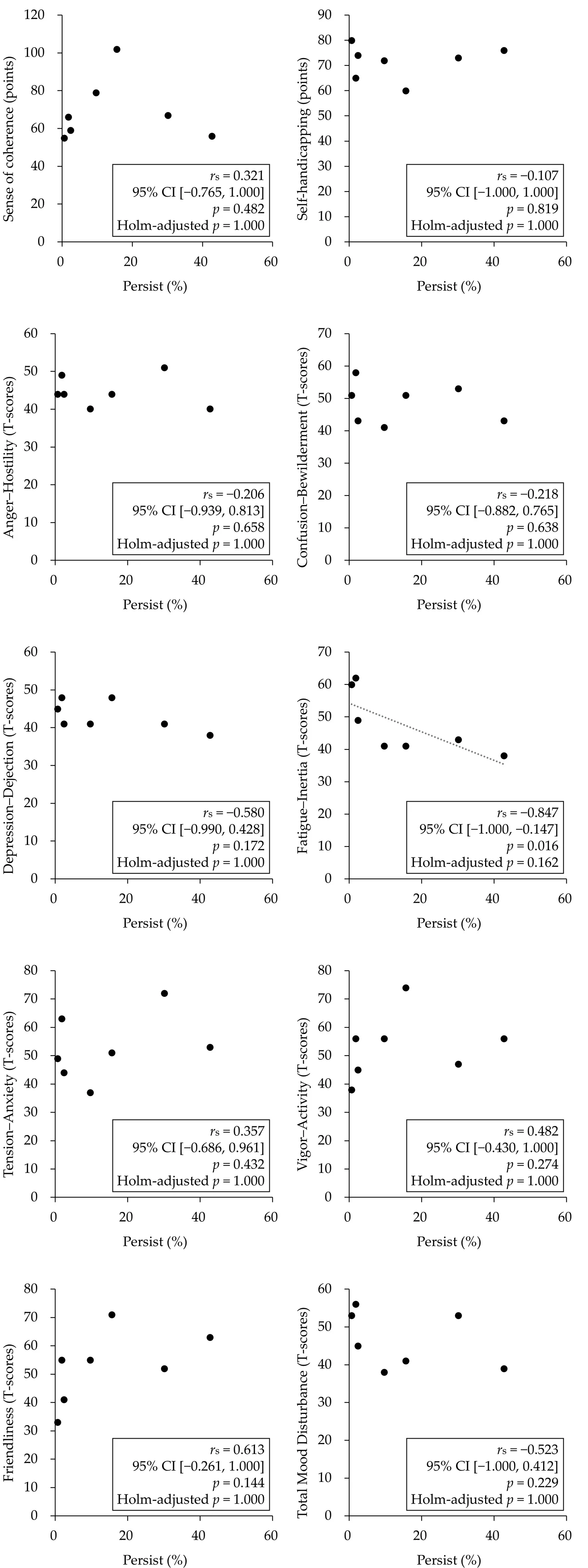
Associations between Persist and questionnaire scores. Dots represent individual participants. 95% confidence intervals for Spearman’s rank correlation coefficients were calculated using 10,000 participant-level bootstrap resamples with replacement using the percentile method. Adjusted *p*-values were calculated using the Holm procedure. The dashed line is shown for Fatigue–Inertia to visualize the negative association observed in the unadjusted analysis; however, this association did not remain statistically significant after Holm correction.

The relationships between Persist and each questionnaire score are shown in Figure 1. Persist showed a negative association with FI in the unadjusted analysis (*r*_s_ = −0.847, 95% CI [−1.000, −0.147], *p* = 0.016); however, this association did not remain statistically significant after Holm correction for multiple comparisons (adjusted *p* = 0.162). No other associations were statistically significant in either the unadjusted or Holm-adjusted analyses.

In the leave-one-out sensitivity analysis, the association between Persist and Fatigue–Inertia remained negative in all seven iterations (*r*_s_ range: −0.928 to −0.754), whereas the corresponding unadjusted *p*-values ranged from 0.008 to 0.084. Thus, the direction of the association was consistent, but statistical significance was not robust to the exclusion of individual participants.

## 4. Discussion

In this exploratory field study, a higher weekly Persist proportion was negatively associated with POMS 2 Fatigue–Inertia in the unadjusted analysis; however, this association did not remain statistically significant after Holm correction. Accordingly, the observed association should be regarded as preliminary and hypothesis-generating. Several interpretations of this association are possible, and the present observational design does not establish either its causal direction or its underlying mechanism.

Athletes with lower fatigue-related mood states may have voluntarily undertaken more high-intensity training, whereas athletes experiencing greater fatigue may have reduced their training intensity. Differences in fitness, session content, rest periods, recovery status, and total training duration may also have influenced Persist independently of fatigue-related mood states. Because Persist is expressed as a proportion of total training time, variation in the denominator may additionally affect the observed association.

POMS 2 Fatigue–Inertia is conceptually distinct from experimentally induced mental fatigue. Experimental and systematic-review evidence suggests that experimentally induced mental fatigue may influence perceived exertion and physical or sport-specific psychomotor performance [16,17,18], whereas a bias-sensitive meta-analysis has questioned the magnitude and robustness of these effects [19]. Recent field-based research has also explored whether routinely collected external-load data contain fatigue-related information, illustrating both the potential and methodological challenges of ecological monitoring approaches [20].

In kendo specifically, previous studies have examined attention- and motivation-related neural activity [21], perception–action processes involved in interpersonal distance regulation [22], and psychological characteristics of kendo practitioners [23]. Changes in urinary serotonin during kendo competition involving mental stress have also been reported [24]. These findings provide sport-specific context for considering psychological and perceptual factors in kendo but do not provide mechanistic evidence for the association observed in the present study.

From an applied perspective, Persist represents an individualized wearable-derived measure based on the proportion of training time spent above an RPE-anchored HR threshold. Whether it provides information beyond established training-load measures or questionnaire-based assessments remains unknown. No significant associations were observed between Persist and SOC or SHC; however, given the very small sample, these null findings should not be interpreted as evidence that Persist is independent of these psychological traits. Persist therefore primarily quantifies the proportion of training performed above an individualized HR threshold and may reflect not only fatigue-related mood states but also training-intensity distribution, session content, fitness, recovery status, motivation, and other factors affecting exercise behavior. Future studies should determine whether repeated assessment of Persist provides practically useful information for athlete monitoring.

This study has several limitations. First, the sample size was small and included only female athletes, which limits the precision and generalizability of the findings and precludes examination of possible sex differences. In addition, ten correlations were examined, and the observed association between Persist and Fatigue–Inertia did not remain statistically significant after Holm correction for multiple comparisons; therefore, this finding should be considered hypothesis-generating. Second, the observational design does not allow causal relationships to be determined. In addition, fatigue-related mood states were assessed using a single retrospective POMS 2 assessment referring to the preceding week, and no repeated within-athlete mood assessments were obtained. Therefore, the present study cannot determine whether within-athlete changes in Persist correspond to changes in fatigue-related mood states over time. Third, training was neither standardized nor systematically characterized because participants followed their own training plans. Differences in training content, session structure, rest periods, intensity distribution, fitness, and recovery status may therefore have influenced Persist independently of fatigue-related mood states. Accordingly, the observed association cannot be attributed specifically to fatigue-related mood states. Fourth, the individualized HR threshold was derived during cycle ergometry and subsequently applied to field-based kendo training. The preliminary exercise test was performed only once; therefore, the test–retest reliability and day-to-day stability of the individualized RPE 13 HR threshold were not assessed. In addition, the validity of transferring the RPE 13–HR relationship from cycle ergometry to field-based kendo training was not established. Fifth, HR during kendo training was measured using a wrist-worn photoplethysmographic device. The device was worn on the left wrist to minimize direct impact from the shinai, and no missing HR epochs were identified. Nevertheless, rapid upper-limb movements and gripping may introduce motion artifacts, and no concurrent reference HR measurement was obtained during field monitoring. Consequently, possible measurement error or misclassification of time spent above the individualized HR threshold cannot be completely excluded. Finally, whether Persist provides information beyond established measures such as mean HR, time in conventional HR zones, session-RPE, or other internal and external training-load indices remains unknown. Future studies should directly compare Persist with established measures and evaluate its criterion, construct, convergent, and incremental validity.

## 5. Conclusions

In this exploratory field study of seven elite female kendo athletes, Persist showed a negative unadjusted association with POMS 2 Fatigue–Inertia (*r*_s_ = −0.847, *p* = 0.016), but the association was not statistically significant after Holm correction (adjusted *p* = 0.162). Given the small sample, observational design, uncontrolled training content, and potential measurement and training-load confounding, this finding should be considered hypothesis-generating. Larger studies using repeated within-athlete assessments, carefully characterized training, and direct comparison with established training-load measures are required before the utility of Persist for athlete monitoring can be established.

## Figures and Tables

**Table 1:** Physical and exercise-related characteristics of the participants.

Category	Variable	Mean ± SD
Basic characteristics	Age (years)	34.6 ± 5.5
	Height (cm)	166.1 ± 4.5
	Body weight (kg)	64.3 ± 6.9
	Fat-free mass (kg)	46.7 ± 3.3
	Body fat percentage (%)	27.0 ± 5.0
Exercise-related characteristics	Resting HR (bpm)	74.7 ± 8.7
	Maximum HR (bpm)	178.4 ± 5.2
	HR at the point perceived as “somewhat hard” (bpm)	149.0 ± 12.9
	VO_2_max (mL/kg/min)	37.9 ± 5.1
	Resting blood lactate concentration (mmol/L)	1.7 ± 0.5
	Post-test blood lactate concentration (mmol/L)	11.3 ± 3.1

**Table 2:** POMS 2 T-Scores, Sense of Coherence, and Self-Handicapping scores.

Questionnaire	Category	Mean ± SD
POMS 2	Anger–Hostility	44.6 ± 4.2
	Confusion–Bewilderment	48.6 ± 6.3
	Depression–Dejection	43.1 ± 3.9
	Fatigue–Inertia	47.7 ± 9.7
	Tension–Anxiety	52.7 ± 11.7
	Vigor–Activity	53.1 ± 11.5
	Friendliness	52.9 ± 12.8
	Total Mood Disturbance	46.4 ± 7.5
Sense of Coherence		65.4 ± 9.5
Self-Handicapping		71.4 ± 6.8

POMS 2, Profile of Mood States 2nd Edition.

## Data Availability

The data presented in this study are available on request from the corresponding author due to privacy and ethical restrictions.

## Abbreviations

The following abbreviations are used in this manuscript:

POMS 2	Profile of Mood States 2nd Edition
RPE	Rating of Perceived Exertion
HR	Heart Rate
FI	Fatigue–Inertia
SOC	Sense of Coherence
SHC	Self-Handicapping
AH	Anger–Hostility
CB	Confusion–Bewilderment
DD	Depression–Dejection
TA	Tension–Anxiety
VA	Vigor–Activity
F	Friendliness
TMD	Total Mood Disturbance
SOC-13	13-item Sense of Coherence Scale
SD	Standard Deviation
*r*_s_	Spearman’s rank correlation coefficient
VO_2_max	Maximal Oxygen Uptake
